# The tyrosine kinase inhibitor Gefitinib reduces *C. elegans* stress-induced sleep, but not likely via LET-23/EGFR inhibition

**DOI:** 10.17912/micropub.biology.001138

**Published:** 2024-03-12

**Authors:** Caroline Coto, Adrian Arimie, Jesse G Jones, Cheryl Van Buskirk

**Affiliations:** 1 Biology, California State University, Northridge

## Abstract

The anticancer drug Gefitinib is a tyrosine kinase inhibitor with selectivity for the Epidermal Growth Factor Receptor (EGFR/ErbB1). As the *C. elegans* EGF receptor LET-23 shares notable structural homology over its kinase domain with human EGFR, we wished to examine whether Gefitinib treatment can interfere with LET-23-dependent processes. We show that Gefitinib disrupts *C. elegans* stress-induced sleep (SIS) but does not impact EGF overexpression-induced sleep nor vulva induction. These findings indicate that Gefitinib does not interfere with LET-23 signaling and impairs SIS through an off-target mechanism.

**Figure 1. Gefitinib-treated animals show reduced SIS, but not other signs of LET-23/EGFR inhibition:**
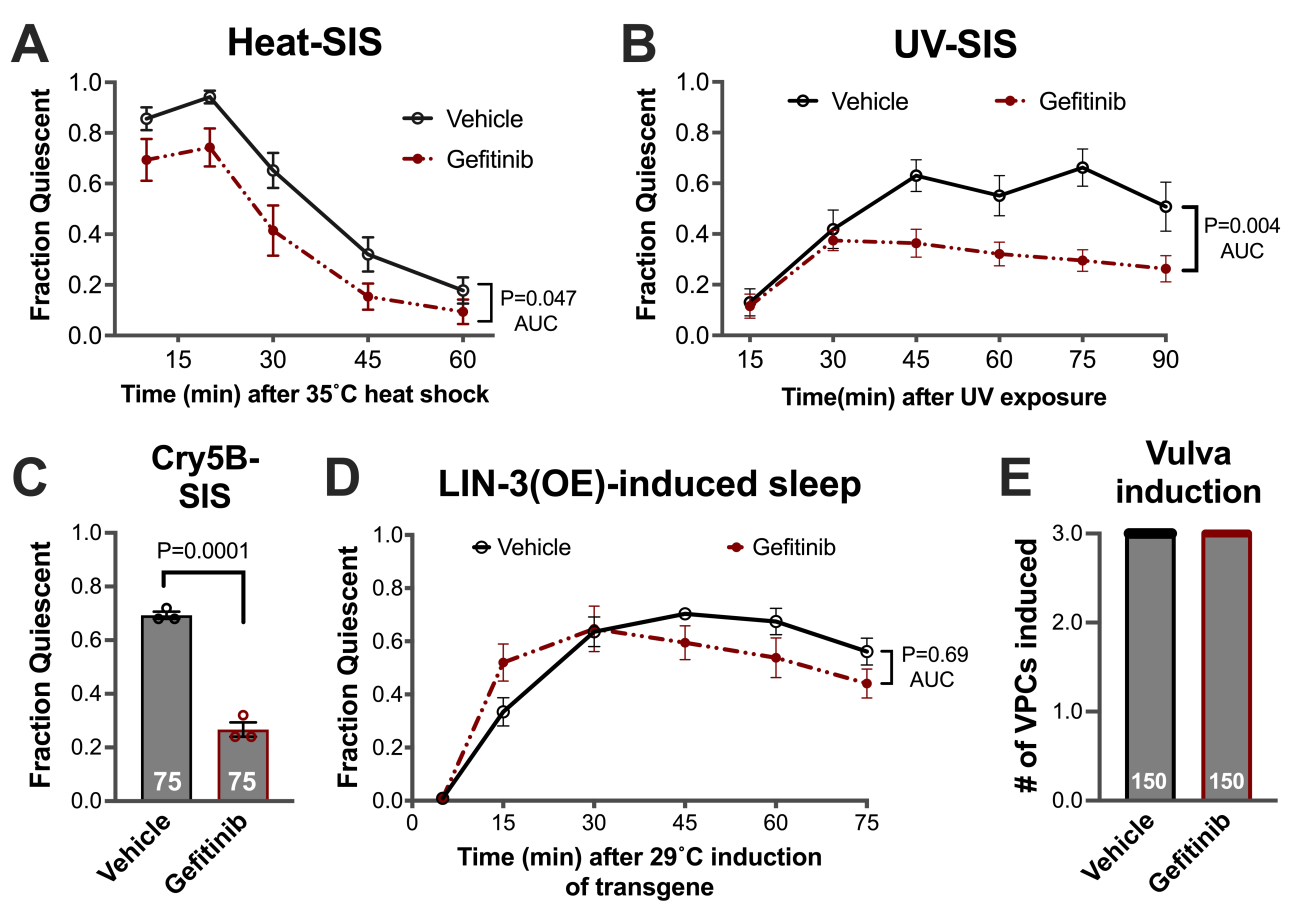
Wild type N2 animals were exposed from hatching to either 10 uM Gefitinib or an equivalent concentration of DMF vehicle and examined for defects in EGFR-dependent processes. (A–C) Young adult animals were assayed for stress-induced sleep (SIS) as described in methods. Gefitinib-treated animals show reduced quiescence relative to vehicle controls during (A) heat-induced sleep, (B) UV-induced sleep, and (C) Cry5B toxin-induced sleep. (D) Young adult animals were assayed for the sleep-like state triggered by ubiquitous overexpression (OE) of the EGF family ligand LIN-3. Gefitinib-treated animals show no defect in LIN-3(OE)-induced sleep. (E) Vulval induction was examined in Gefitinib and vehicle-treated animals at the L4 stage. All animals showed vulval patterning indicative of three VPCs having adopted a vulval fate. At least three trials of 25 animals were performed in A-D. In C and E, the number of animals examined is shown at the base of each bar. P values were determined by unpaired t tests, AUC = area under the curve.

## Description

Receptor tyrosine kinases (RTKs) that respond to the Epidermal Growth Factor (EGF) family of ligands play important roles in development and physiology across metazoa, and dysregulation of EGF receptor (EGFR/ErbB) signaling is associated with several human cancers (Uribe et al. 2021) . The tyrosine kinase domain of the human EGFR serves as the target of several anticancer drugs including Gefitinib , sold under the brand name Iressa (Vansteenkiste 2004) . This tyrosine kinase inhibitor (TKI) reversibly competes with ATP at a critical ATP-binding site, blocking receptor activation (Barker et al. 2001) . We wished to examine whether Gefitinib might inhibit the kinase activity of a distantly related EGFR, namely *C. elegans* LET-23 . The kinase domains of LET-23 and human EGFR show 44% sequence identity (Bogdan and Klämbt 2001), as well as remarkable structural conservation (Liu et al. 2018) . LET-23 plays roles in cell specification events that influence viability and vulval development (Aroian et al. 1990) , as well as physiological processes that influence sleep and aging (Bray 2015; Yu and Driscoll 2011) . As *let-23* null mutants are inviable, genetic analysis of this signaling pathway is limited to the use of partial reduction-of-function alleles, and we are therefore particularly interested in the potential use of Gefitinib as a LET-23 inhibitor. This project was initiated in the undergraduate course BIOL447:FIRE (Full Immersion Research Experience) at California State University, Northridge.

To examine the potential inhibitory effect of Gefitinib on LET-23 , we first examined LET-23-dependent sleep as a measure of LET-23 function. Upon exposure to damaging conditions, *C. elegans* enters a behaviorally quiescent state (Hill et al. 2014) . This stress-induced sleep, or SIS, is dependent on LET-23 activation within sleep-promoting neurons (Hill et al. 2014; Nelson et al. 2014; Konietzka et al. 2020) . We grew animals in the presence of 10 uM Gefitinib or DMF vehicle from hatching and assayed SIS at the young adult stage. We examined three known SIS triggers - noxious heat, exposure to UV light, and ingestion of pore-forming Cry5B toxin (Hill et al. 2014; DeBardeleben et al. 2014) . In each case, we found the Gefitinib-treated animals to be SIS-defective relative to vehicle-treated controls ( Fig. 1 A -C), raising the possibility that Gefitinib interferes with LET-23 tyrosine kinase activity. We note that the Gefitinib-treated animals did not display hyperactivity, which is known in some cases to override the SIS response (for example, Soto et al. 2019).

To examine this further we assayed the LET-23 *-* dependent sleep-like state that can be triggered by overexpression of LIN-3 from a heat-responsive promoter (Van Buskirk and Sternberg 2007) . We used a mild heat shock (29˚C, 30 min) to induce transgene expression, as this condition does not trigger heat-SIS (Nelson et al. 2014) but is sufficient to induce a moderate bout of transgene-dependent sleep. We found that Gefitinib-treated animals exhibit the same amount of LIN-3 (OE) sleep as vehicle-treated controls (D). As LIN-3 (OE) sleep can be suppressed by *let-23 (rf)* mutations as well as by *let-23* RNAi, even under more robust heat shock conditions (Van Buskirk and Sternberg 2007) , these data suggest that Gefitinib does not interfere with LET-23 signaling.

Last, we examined the LET-23-dependent process of vulval precursor cell (VPC) induction. During mid-larval development, LIN-3 from the gonadal anchor cell activates EGF receptors in three nearby VPCs, promoting cell division and patterning that can be readily assayed at the L4 larval stage (Sternberg and Horvitz 1986) . We found that Gefitinib-treated and control animals display vulva morphology consistent with wild-type induction of three VPCs ( Fig. 1E ), indicating that Gefitinib treatment does not impair LET-23 signaling during vulva development.

Together our data indicate that the tyrosine kinase inhibitor Gefitinib impairs *C. elegans* stress-induced sleep but does not interfere with LET-23 activity. As defects in SIS have been observed in animals with enhanced stress responses (Goetting et al. 2020; Kawano et al. 2023) , we posit that Gefitinib exposure may promote stress resistance. Interestingly, Gefitinib has been found to promote reactive oxygen species that contribute to drug resistance in lung cancer cells (Okon et al. 2014) . It is possible that the SIS defects of Gefitinib-treated animals are due to upregulation of stress responses during drug exposure, rendering animals partially resistant to the damaging conditions used to trigger SIS. Our work indicates that while LET-23 is not a Gefitinib target, *C. elegans* SIS might be used to screen TK inhibitors for off-targets with relevance to anticancer drug resistance.

## Methods

Gefitinib (VWR 103823-390) was dissolved 20mg/ml in Dimethylformamide (DMF) and diluted 1/100 in OP50 *Escherichia coli* bacteria that had been concentrated 20x from liquid culture by centrifugation. 100 ul of this 'thick' OP50 solution containing 200 ug/ml Gefitinib (or an equivalent volume of DMF as a control) was used to seed 5 ml nematode growth media (NGM) plates for a final plate concentration of 4 ug/ml (10 uM) Gefitinib. This is twice the Gefitinib concentration used previously to inhibit the EGFR tyrosine kinase activity of a chimeric LET-23::hEGFR expressed in *C. elegans* (Bae et al. 2012) and presumably enters the animals via ingestion, as the cuticle is relatively impermeable. Vehicle control plates contained an equivalent volume (0.04%) of DMF. We used thickened OP50 so that eggs could be added to the dried bacterial lawn 4-5 hours later. For SIS and vulva induction assays, wild type N2 eggs were added to plates and cultivated at 20˚C. In all sleep assays, animals were scored with experimenter blind to treatment, and sleep was defined as a complete cessation of locomotion and feeding during a 3 sec observation at each time point. At least 3 trials were performed for each sleep assay, with at least 25 animals per trial per treatment.

**Heat-SIS** : Young adult animals were transferred to small (35 X 10 mm, 5 ml) NGM plates seeded with OP50 and sealed with parafilm. Plates were positioned lid-up in a 35°C water bath for 30 min and placed at 4˚C for 1 min to cool them to room temperature.

**UV-SIS** : Young adult animals were transferred to small NGM plates seeded with OP50 and positioned lid-side down on a 302 nm (UVB) transilluminator gel box for 50 sec. The same lid was used for all samples in a given trial, to control for potential variability in lid thickness.

**Cry5B-SIS** : Young adult animals were placed for 10 min onto NGM plates containing 60 μg/mL carbenicillin and 1 mM IPTG that had been seeded with JM103 bacteria harboring an IPTG-inducible Cry5B toxin. After exposure, animals were transferred to NGM plates seeded with OP50 *E.coli* for another 10 min and assayed for sleep at a single time point.

**Vulva induction** : L4 larval animals were examined by differential interference contrast (DIC) microscopy, assaying vulval cells and lumen for deviations from the symmetric tree-like appearance characteristic of wild-type vulval cell induction and morphogenesis.

**LIN-3 overexpression** : Animals harboring the *syIs197* [hs: LIN-3 ; *myo-2* :dsRED] transgene (Van Buskirk and Sternberg 2007) were exposed to Gefitinib or vehicle from hatching and heat-shocked at the young adult stage as described above but at 29˚C instead of 35˚C, with the cooling step omitted.

**Statistical Analysis** : Data was graphed and analyzed as described in the figure legend, using GraphPad Prism software.

Strain Table:

**Table:** 

**Strain**	**Genotype**	**Access**
N2	Wild type	CGC
PS5970	*him-5(e1490)* *syIs197* [hs::LIN-3c(cDNA) + *myo-2* p::DsRed + *pha-1(+)* ] V	CGC
